# Estrogen and Cardiovascular Health

**DOI:** 10.3389/fcvm.2022.886592

**Published:** 2022-03-30

**Authors:** Hester M. den Ruijter, Georgios Kararigas

**Affiliations:** ^1^Laboratory of Experimental Cardiology, University Medical Center Utrecht, Utrecht, Netherlands; ^2^Department of Physiology, Faculty of Medicine, University of Iceland, Reykjavík, Iceland

**Keywords:** cardiac, heart failure, steroid hormone, vasculature, transgender

## Introduction

The steroid hormone 17β-estradiol (E2), together with its receptors (ER), is thought to play a major role in the modulation of cardiovascular physiology and pathology (1–6). E2 signals through the classical nuclear ERα and ERβ, as well as the membrane G protein-coupled receptor GPR30 (also referred to as GPER), via the genomic or non-genomic pathway. The E2/ER axis has been shown to exert vast effects in the cardiovascular system, regulating contractile function, (micro)vascular function, metabolic processes, calcium signaling, gene expression and protein abundance (7–22), among others, which can be sex-dependent (3, 23–28).

**Figure 1:**
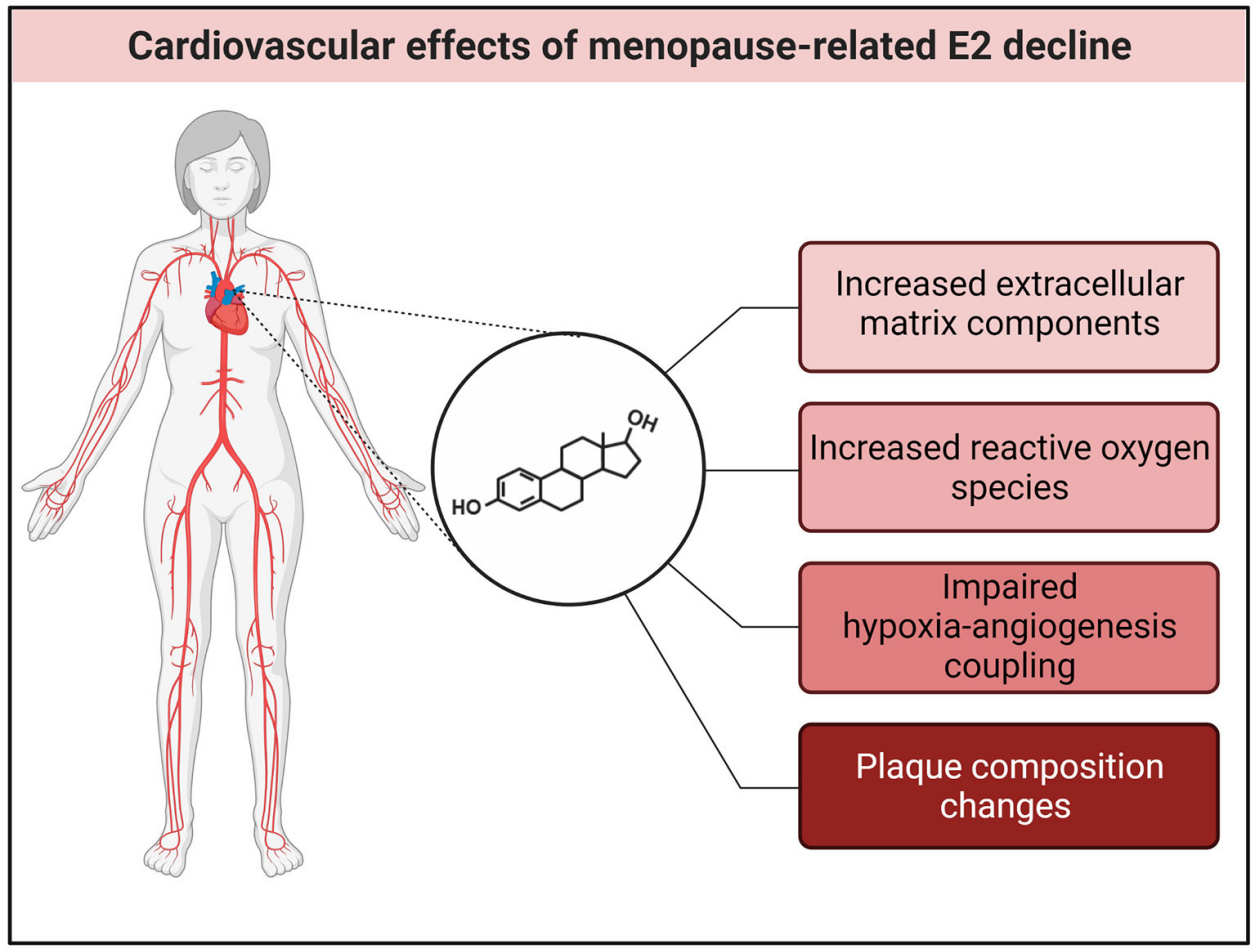
E2 effects in the cardiovascular system. The decline of E2 at menopause may lead to an increase in extracellular matrix (ECM) components and reactive oxygen species (ROS), impaired hypoxia-angiogenesis coupling and increased atherosclerotic plaque formation. Created with BioRender.com.

From a clinical perspective, the decline in E2 at menopause may contribute to the onset of cardiovascular disorders, such as atherosclerosis, heart failure with preserved ejection fraction (HFpEF) and other conditions that involve the microvasculature (9, 29, 30). Along this line, a consensus article was published recently by Maas et al. (31), which we found very informative and clear, and the authors are to be commended. The authors discuss several biological processes and pathways, which may eventually impact cardiovascular health and risk factors. These include altered vascular function, enhanced inflammation and up-regulation of other hormonal systems, such as the renin-angiotensin-aldosterone system, the sympathetic nervous system, and reduced nitric oxide-dependent vasodilation, as well as vascular and myocardial stiffness. Some additional key mechanisms require consideration and are briefly highlighted in this article (Figure 1).

## E2 Mechanisms in the Cardiovascular System

Cardiovascular remodeling and dysfunction are associated with alterations and disruptions of homeostasis of the extracellular matrix. Vascular and myocardial stiffness has been widely associated with fibrillar collagen and cross-linking. Given that E2 is involved in the regulation of collagens (32), decreased E2 levels might eventually contribute to increases in cardiac extracellular matrix components in postmenopausal women (33).

Endothelial dysfunction is associated with increased systemic oxidative stress and vascular inflammation, characterized by reduced vasodilators, such as nitric oxide. Antioxidant properties of E2 have been previously reported, scavenging free radicals, as well as inhibiting the formation of mitochondrial reactive oxygen species, thereby leading to lower levels of oxidative stress (34, 35). Reduced E2 levels in postmenopausal women may also contribute to an impaired hypoxia-angiogenesis coupling, thereby limiting the recovery from ischemia.

Interestingly, recent work on human atherosclerotic plaques suggests that E2 may also regulate gene expression of multiple downstream targets and thereby influence the activity of molecular networks, even years after menopause (36). In postmenopausal women undergoing coronary artery bypass grafting, atherosclerotic plaques showed enrichment of the gene set E2 responses in molecular networks important for smooth muscle cells of the plaque (36).

## E2 in Transgender Women

In the article by Maas et al., we were also very interested to see the discussion on cardiovascular disease risks for cross-sex therapy in transgender women (31). The authors point to the fact that only venous thromboembolism risk has been evaluated in transgender women undergoing estrogen treatment. This clearly highlights an important gap in the field. They further note that transgender women have an increased risk for various cardiovascular disorders. This is not surprising. E2 is primarily synthesized in the gonads, particularly in the ovaries. However, there are extra-ovarian sources of E2 production. These include the adipose, breast and adrenal tissues, bone, heart, brain and skin, where aromatase can be produced (28). In addition, the testes and prostate are production sites of E2 through the local conversion of androgenic precursors by the aromatase enzyme, which may lead to significant levels of E2. In this context, there may be a marked increase of E2 production in individuals with obesity (37), as the adipose tissue is a major contributor of E2 synthesis, and in elderly individuals, men may have higher concentrations of E2 compared with age-matched women (38). Notably, elevated E2 levels have been associated with an increased risk and incidence of cardiovascular disease in cisgender men (39–41). Although explanations for causal pathways and putative mechanisms for this association are incompletely understood, E2 appears to influence the contractile machinery, modulating regulatory proteins (24).

## Conclusions

Collectively, we greatly appreciate the focus of the article by Maas *et al*. The elucidation of underlying mechanisms is essential to the identification of potential therapeutic targets with the ultimate goal of improving medical care. Clearly, further research into the effects of menopause-related decreases in E2 on cardiovascular (patho)physiology is warranted.

## Author Contributions

All authors have read and agreed to the published version of the manuscript.

## Funding

GK acknowledges lab support provided by grants from the Icelandic Research Fund (217946-051), Icelandic Cancer Society Research Fund and University of Iceland Research Fund.

## Conflict of Interest

The authors declare that the research was conducted in the absence of any commercial or financial relationships that could be construed as a potential conflict of interest.

## Publisher's Note

All claims expressed in this article are solely those of the authors and do not necessarily represent those of their affiliated organizations, or those of the publisher, the editors and the reviewers. Any product that may be evaluated in this article, or claim that may be made by its manufacturer, is not guaranteed or endorsed by the publisher.
